# Medication of the Respiratory Passages

**Published:** 1857-12

**Authors:** 


*Medication of the Respiratory Passages.* (Académie de Médecine, Paris, August 25, 1857.) Translated by JAMES JONES, M. D.

Dr. Trousseau read a report on a memoir of Dr. Loiseau, entitled: *A simple and easy process whereby to penetrate into the respiratory passages in order to cauterizé them,* to extract false membranes, to dilate the glottis, and to introduce all the pulverulent or liquid substances when not acceptable.

The reporter stated that the number of cures obtained through tracheotomy, had removed in late years the species of interdiction which was laid on that operation. Nevertheless, it did not prevail among the mass of physicians, and is only practised by a small number. The majority discountenance it either on account of its difficulty, or the insufficient proof of its utility.

After having seen many fatal cases of croup in which tracheotomy, although indicated, was not performed, Dr. Loiseau has conceived certain instruments and a practical method for penetrating into the larynx as we do into the pharynx. This invention dates from the year 1840.

Dr. Green, of New York, had already suggested to reach the interior of the larynx by a stiff whalobone with a curve terminated by a small sponge, of which the introduction was facilitated by a tongue-depressor, which in his hands was a good instrument. It forms a large spatula, concave beneath, with a horizontal part for the tongue and the handle at a right angle, it can be held without masking the interior of the mouth and thus completely exposes the epiglottis. Nevertheless, it is very difficult to penetrate between the arytæno-epiglottic folds with the end of the whalebone, by reason of their spasmodic contraction, and even on the dead body we succeed only one time in four.

The process of Loiseau is more simple and it is infallible. He protects the base of the left forefinger by a metallic ring of about an inch in height, and introduces it rapidly and far back into the mouth, so that the ring is placed between the molar teeth and holds the jaws open. With the free extremity of the finger at the same time that he depresses the tongue, he seizes tne epiglottis, raises it, and shoves the pulp of the index between the folds. Nothing is then easier than to slide a laryngeal tube along the finger, which tube is no other than Chaussier's. The escape of air from the end of the tube proves its entrance into the larynx. It only remains then to apply by means of this tube as a conductor, either the nitrate of silver or any other substance contained in a little cavity in the side of a flexible metallic rod. In substituting the forceps for the laryngeal tube, we can by a similar process extract foreign bodies which have slipped into the air passages.

In May, 1839, Dieffenbach having used the same method, employed in the identical style, in a case of croup, to him belongs the priority. The reporter believes that Loiseau was ignorant of this fact, which, not having been published, only came by accident to the knowledge of the committee.

In another part of his memoir, the author prefers the employment of tannin in membraneous angina to the exclusion of cauterization, which treatment has met with uniform success in *in many hundred cases* observed by himself. Dr. Trousseau having recalled to mind that Areteus had already boasted of gall-nuts and tannin in the Egyptian and Syrian ulcers, which are nothing but diptheritis, demanded if in some of his cases, Loiseau had not confounded membranous angina with that disease. He had seen Loiseau's practice succeed, but could not consent to abandon caustics.

As to Loiseau's operation, recommended previously by Reyband—which consists in leaving a canula permanently in the glottis—he condemns it as barbarous and impracticable.

In conclusion, he thinks that the method of catheterizing the larynx in this memoir of Loiseau, is a good substitute for tracheotomy, and ought in all cases to be used before this operation. He proposed, therefore, to thank the author and to publish his work.—*L' Union Médicale,* from *New Orleans Med. & Surg. Journal.*

